# Frequency and time-frequency analysis of intraoperative ECoG during awake brain stimulation

**DOI:** 10.3389/fneng.2013.00001

**Published:** 2013-02-25

**Authors:** Emanuela Formaggio, Silvia F. Storti, Vincenzo Tramontano, Agnese Casarin, Alessandra Bertoldo, Antonio Fiaschi, Andrea Talacchi, Francesco Sala, Gianna M. Toffolo, Paolo Manganotti

**Affiliations:** ^1^Department of Neurophysiology, Foundation IRCCS San Camillo HospitalVenezia, Italy; ^2^Clinical Neurophysiology and Functional Neuroimaging Unit, Department of Neurological, Neuropsychological, Morphological and Movement Sciences, Section of Neurology, University HospitalVerona, Italy; ^3^Department of Neurological, Neuropsychological, Morphological and Movement Sciences, Section of Neurosurgery, University HospitalVerona, Italy; ^4^Department of Information Engineering, University of PadovaPadova, Italy

**Keywords:** ECoG, bipolar, monopolar, relative wavelet power, AR model

## Abstract

Electrocortical stimulation remains the standard for functional brain mapping of eloquent areas to prevent postoperative functional deficits. The aim of this study was to investigate whether the short-train technique (monopolar stimulation) and Penfield's technique (bipolar stimulation) would induce different effects on brain oscillatory activity in awake patients, as quantified by electrocorticography (ECoG). The study population was seven patients undergoing brain tumor surgery. Intraoperative bipolar and monopolar electrical stimulation for cortical mapping was performed during awake surgery. ECoG was recorded using 1 × 8 electrode strip. Spectral estimation was calculated using a parametric approach based on an autoregressive model. Wavelet-based time-frequency analysis was then applied to evaluate the temporal evolution of brain oscillatory activity. Both monopolar and bipolar stimulation produced an increment in delta and a decrease in beta powers for the motor and the sensory channels. These phenomena lasted about 4 s. Comparison between monopolar and bipolar stimulation showed no significant difference in brain activity. Given the importance of quantitative signal analysis for evaluating response accuracy, ECoG recording during electrical stimulation is necessary to characterize the dynamic processes underlying changes in cortical responses *in vivo*. This study is a preliminary approach to the quantitative analysis of post-stimulation ECoG signals.

## Introduction

Cortical electrical stimulation is a complex neurophysiological brain mapping technique by which an electrical current is directly applied to the cortex to induce temporary, local cortical activation or disruption. In common use for over seven decades in the surgical treatment of medically refractory epilepsy (Penfield and Jasper, 1954; Marson, 1980) and brain tumors, it creates a real-time functional map of the brain surface according to which a safe boundary for tumor resection can be delineated (Van Buren et al., 1978; Gregorie and Goldring, 1984; Berger et al., 1989; Ojemann et al., 1989; Ebeling et al., 1992; Berger, 1995; Berger and Rostomily, 1997; Sawaya et al., 1998; Duffau et al., 1999; Taylor and Bernstein, 1999; Ebel et al., 2000; Sahjpaul, 2000; Bernstein, 2001; Meyer et al., 2001).

In neurosurgery, electrical stimulation remains the standard for functional brain mapping of eloquent areas to prevent postoperative functional deficits. Although considered the gold standard, electrocortical stimulation mapping methodologies vary across studies and institutions. Many questions regarding its mechanisms remain unanswered. The basic principle of cortical stimulation relies on the application of an electrical impulse on the cortex. Two different methods have been established: short-train technique and Penfield's technique.

The short-train technique, usually performed using a monopolar stimulating probe, derived from investigations by Hern et al., is a proven and reliable method for monitoring subcortical pathways (Hern et al., 1962). It is as sensitive as Penfield's technique for mapping the primary motor cortex (Cedzich et al., 1996) but it requires a lower stimulation intensity to trigger a motor evoked potential (MEP) (Gorman, 1996). This widely used method allows monitoring of MEPs during intraoperative monitoring (Szelényi and Deletis, 2004; Deletis, 2005; Szelényi et al., 2007; Talacchi et al., 2010; Deletis and Sala, 2011).

Penfield's technique, introduced by Penfield and Boldrey and later modified by LeRoux et al., is used for mapping the motor cortex, especially the premotor frontal cortex (Penfield and Boldrey, 1937; LeRoux et al., 1991; Kombos and Süss, 2009). The technique accounts for a sustained train of stimuli of 0.5 ms duration, delivered at rate of 60 Hz up to a maximum amplitude of about 18–20 mA by means of bipolar probes. It is still employed today for intraoperative mapping of speech related cortices. The main drawback of this technique is the high incidence of intraoperative seizures induced by prolonged stimulation. As an alternative, low-frequency (5 and 10 Hz) electrical stimulation has been proposed to decrease the risk of afterdischarges (Zangaladze et al., 2008).

The two techniques differ from one another in that short-train stimulation preferentially activates the fibers originating from the primary motor cortex, whereas Penfield's stimulation elicits motor responses when the pre-motor frontal cortex is stimulated (Kombos et al., 1999; Szelényi et al., 2010, 2011). While both methods are equally sensitive for mapping the primary motor cortex, Penfield's technique is more sensitive in localizing cognitive functional areas in the pre-motor frontal cortex but less specific for the motor area. Since short train method activates the corticomotoneural tract, it achieves better results in areas with a high density of pyramidal-cells. Thus, location is better in the primary motor cortex (Kombos et al., 1999).

Electrocorticography (ECoG), a neurophysiological technique to record cortical potentials from the exposed brain in the operating room, records the same type of cerebral potentials as the scalp electroencephalogram (EEG), but with less dispersion and attenuation of the potential due to the absence of scalp and skull. It is useful for continuous monitoring during stimulations and for afterdischarge activity in the absence of physical signs of seizure (non-convulsive seizure) which can occur after electrical stimulation of the cortical areas (Zangaladze et al., 2008). In operative standard evaluation, the ECoG is visually inspected by the physician without quantitative measures.

In electroneurophysiological analysis, spectrograms obtained via Fourier transform (FT) or autoregressive models (AR) are used to assess the frequency content of electrophysiological activity. If a signal contains frequency components that emerge and vanish within certain time intervals, as after an electrical stimulation, time as well as frequency information is required. These methods are usually based on the assumption that the data are stationary. However, processing of information by the brain is reflected in dynamical changes of electrical activity over time, frequency, and space. Therefore, to study this process, methods are needed which can describe signal variation in time and frequency simultaneously. There is increasing interest in the use of wavelet-based techniques for processing non-stationary EEG recordings not only with respect to oscillatory behavior (Klein et al., 2006) but also for spike detection (Senhadji and Wendling, 2002), sleep stage identification (Jobert et al., 1994) and filtering (Glassman, 2005). In addition to providing spectral statistics similar to those obtained with FT or AR, wavelet-based methods can detect temporal evolution. Specifically, relative wavelet power (RWP) provides information about the relative power associated with the different frequency bands present in ECoG.

The aim of this study was to investigate whether the monopolar short-train technique and bipolar Penfield's technique in awake patients would induce different effects on brain oscillatory activity. To quantify the electrical changes recorded on ECoG, we applied a frequency and a time-frequency analysis to compare ECoG relative powers after both stimulation methods. To our knowledge, there are no studies describing the effects of intraoperative electrical stimulation on human brain oscillatory activity. This study is a preliminary approach to the quantitative analysis of post-stimulation ECoG signals. By quantifying the physiological effects of electrical stimulation, this new method could find use in clinical neurophysiology and in the clinical evaluation of the efficacy of both stimulation techniques. In particular, this study highlights the possibility to analyze changes of oscillatory activity induced by electrical stimulation, comparing the results with that obtained using non-invasive brain stimulation such as transcranial magnetic stimulation (TMS). Moreover, increasing methodological aspect this study could have also a clinical impact. A possible application could be the real-time analysis. In this case, the results can be assessed immediately, in real-time, and repeated if ambiguous. Moreover, because stimulation can induce seizures, a medical risk to the patient, a real-time analysis is in some sense safer, as we are able to identify in advance any electrical modification of the brain.

## Materials and methods

### Patients

The study protocol and informed consent documents were approved by the Ethics Committee of Verona University Hospital. All patients provided their informed consent prior to entering the study. The study population was seven patients (4 males and 3 females; mean age, 54±19.9 years; range, 36–85) undergoing brain tumor surgery for: glioma (*n* = 4); meningioma (*n* = 1); and cavernous angioma (*n* = 2) (Table 1). Since patient guidance is essential and each step of the stimulation procedure is announced, the patients were awake after craniotomy. Anesthesia was induced with bolus doses of propofol 0.8 mg Kg^−1^h^−1^, remifentanil 0.01 gamma Kg^−1^ min^−1^ totally endovenous and 5 mg midazolam (benzodizepine) as co-adjuvant. The craniotomy was performed while the patient was under local anesthesia. During cortical mapping the propopofol was suspended and the remifentanil was maintained at 0.01 gamma Kg^−1^min^−1^. As the patients were carefully examined and asked about sensation, feelings, or movements, they had to be awake and cooperative with a full level of consciousness. After tumor surgery, propofol was increased from 0.8 mg Kg^−1^h^−1^to 1.5 mg Kg^−1^h^−1^and remifentanil from 0.01 gamma Kg^−1^ min^−1^to 0.04 gamma Kg^−1^ min^−1^.

**Table 1:** **Tumor type and localization**.

**Patient**	**Side**	**Tumor depth**	**Location**	**Histology**
1	Left	Cortical—subcortical	Rolandic	GBM
2	Left	Cortical—subcortical	Rolandic	GBM
3	Left	Subcortical	Gyrus cinguli	GBM
4	Left	Cortical—subcortical	Rolandic	MENING
5	Left	Subcortical	Rolandic	ANG CAV
6	Left	Cortical—subcortical	Rolandic	ANG CAV
7	Left	Cortical	Insular	ASTROCYT

GBM, glioblastoma IV °; MENING, meningioma; ANG CAV, cavernous angioma; ASTROCYT, astrocytoma II°.

### Somatosensory evoked potentials (SEPs) and phase reversal

**Figure 1:**
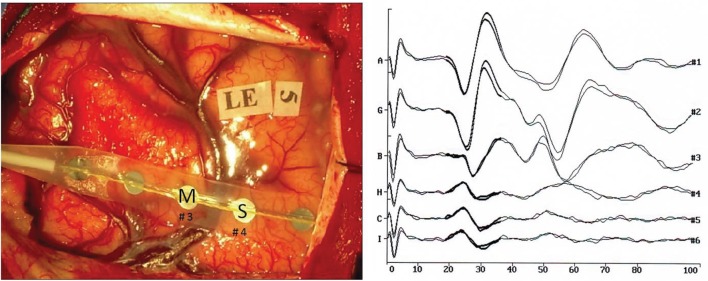
**(Left)** Intraoperative photograph of the brain surface showing electrode placement. M indicates the primary motor cortex, S the primary sensory cortex. **(Right)** Recordings of somatosensory evoked responses to contralateral median nerve stimulation (negative up) in patient no. 2. Scale: 40 μV (trace A), 30 μV (traces G, B, H, G, I); Window: 0–100 ms; Sweeps averaged: 100. Phase reversal of N and P is observed between electrodes nos. 3 and 4.

After craniotomy and opening of the dura mater, 1 × 8 electrode strip (Ates Medica, Verona, Italy) were positioned on the brain 1 cm apart from each other to distinguish the precentral gyrus and the postcentral gyrus (motor and sensory areas) (Figure 1, left). The central sulcus was intraoperatively localized with SEPs phase reversal from the stimulation of the contralateral median nerve (Gregorie and Goldring, 1984; Cakmur et al., 1997) (Figure 1–right). The median nerve was stimulated using a Sentinel 4 evoked potential system (Axon Systems, Inc. Hauppauge, NY, USA). Electrode paste was applied to reduce electrical resistance at the contact between the stimulation electrode and the skin. The cathode was placed proximal to the anode. The electrical stimulus was selected as constant-voltage rectangular waves (rate, 4.3 Hz; pulse duration, 0.2 ms) to stimulate the contralateral nerve at the upper limb, and at a rate of 8.1 Hz with the same pulse duration at the lower limb. All recordings were performed using an Fpz electrode as reference, a band-pass filter 30–300 Hz, and a time base of 100 ms. Between 30 and 100 responses were averaged.

### Electrocortical stimulation mapping

Cortical mapping was performed by Penfield's technique (*bipolar stimulation*): single stimuli of 1 ms at 50 Hz [interstimulus interval (ISI) 20 ms] in a biphasic fashion for at least 3–4 s, and short-train technique (*monopolar stimulation*): trains of 5 stimuli of 0.5 ms at 250 Hz (ISI 4 ms) in a monophasic fashion for 1-s; two different probes. The bipolar probe has two gold tips 6–10 mm apart, while the monopolar probe has a single steel plate of 12 mm as the anode and a frontal reference electrode as the cathode placed on the skin. Stimulation intensity never exceeded 10 mA. We recorded MEP by muscles contralateral to the lesion in the upper and lower limbs. While stimulating the motor cortex, we observed the patient for twitches in response to stimulation. The initial current intensity was set at 2 mA. The mapping threshold was defined as the minimum current needed to induce a motor response, as determined by incrementally increasing stimulation currents (in steps of 2 mA) until a response was observed. Both stimulation modes were performed in four patients (nos. 4, 5, 6, and 7), only bipolar stimulation in two patients (nos. 1, 2), and only monopolar in patient no. 3.

### Electrocorticography procedure

ECoGs were recorded using an EEG system (Quick Brain System 98, Micromed, Treviso, Italy) set at a sampling rate of 512 Hz, using a 1 × 8 electrode strip and one electrode of the strip as reference, usually it is the one farthest from the posterior sulcus and not over the primary motor cortex (M1) or the primary somatosensory cortex (S1). The strip was the same one used to find the central sulcus and was placed mediolaterally in the hand area. Usually the electrodes nos. 4 and 5 are placed over the sulcus of the sensory-motor area. The stimulation was applied over the same cortical area within the same patient and it was delivered at about 3 cm from the strip but the exact position depended on the area stimulated (Broca's area or motor area). In this way the recording field and the position of the strip differ among patients since they are related to the individual motor area. The EEG amplifier had a resolution of 22 bits (range, ± 800 μ V). An anti-aliasing hardware band-pass filter was applied (bandwidth, 0.33–134.7 Hz).

### Data analysis

**Figure 2:**
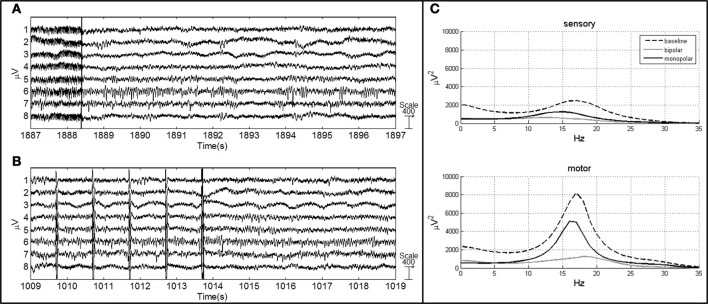
**ECoG of patient no. 4 after bipolar (A) and monopolar (B) stimulation.** The vertical line represents the end of the stimulus **(A)**, vertical lines represent the artifact induced by short-train stimulation and the last line represents the end of the stimulus **(B)**. **(C)** AR power spectrum of sensory and motor channels in three conditions: baseline, after bipolar stimulation and after monopolar stimulation.

ECoG artifacts were identified by visual inspection of the raw signal by a neurologist. Channels affected by electrode artifacts were excluded from subsequent analysis, and individual stimulus response trials were excluded if there was any motion artifact. Post-stimulus time periods were determined by visual inspection of the ECoG recording (Figure 2). The data were processed, maintaining the same reference acquisition, using Matlab 7 (MathWorks, Natick, MA, USA). Frequency and time-frequency analyses were applied to characterize dynamic processes underlying changes in cortical responses.

#### Frequency analysis

Spectral estimation was performed using a parametric approach based on an AR model (Figure 2). ECoG recordings were low-pass filtered at a cut-off frequency of 35 Hz using a finite impulse response (FIR) filter. Epochs were baseline corrected and detrended. The final spectral estimate for each patient was obtained by averaging 20 epochs of 1 s for the baseline condition (ECoG recorded during resting-state condition) and 7 segments of 1 s for the post-stimulus condition (1 s starting 500 ms after stimulus termination). Based on power spectra P(f) (μV^2^), relative powers RP (%) in delta (RP_δ_ 0–4 Hz), theta (RP_θ_ 5–8 Hz), alpha (RP_α_ 9–16 Hz) and beta (RP_β_ 17–32 Hz) frequency ranges were evaluated according to the level decomposition obtained using the time-frequency analysis. The relative powers were normalized at baseline condition to unity in order to compare the power spectra among the patients.

#### Time-frequency analysis

Time-frequency data were assessed by wavelet-based analysis to evaluate the temporal evolution of brain oscillatory activity. A discrete wavelet transform (DWT) was applied to 20 epochs of 1 s for the baseline condition and to 7 epochs of 1 s after stimulus (1 s starting 500 ms after stimulus termination). The wavelet Daubechies 4 (db4) was used as the mother wavelet to generate a family of orthogonal functions (Samar et al., 1999). According to the Mallat algorithm or Mallat-tree decomposition (Mallat, 1998), the DWT was computed by successive low-pass and high-pass filtering of the discrete time-domain signal. We applied the algorithm of decomposition at 6 levels in order to obtain the frequency ranges of interest: delta (0–4 Hz); theta (5–8 Hz); alpha (9–16 Hz); and beta (17–32 Hz). The resulting low frequency subband signals are called approximations, and the high frequency subband signals are called details. For each level, in the wavelet transform, the approximation can be divided into a new approximation and detail subband signal. In each iteration, the highest frequency in the detail band is reduced by half. The relative wavelet powers (RWPs) in the four frequency ranges were computed as described in Rosso et al. (2003). The RWP in baseline condition was obtained by averaging the RWPs of 20 epochs, while the RWPs in post-stimulus condition were computed for each epoch of 1 s (7 values). For both analyses, the responses were normalized for each electrode with respect to a baseline recording prior to stimulation in order to compare the RWPs among the patients.

#### Statistical analysis

Because data were not sampled from a normal distribution and because the number of patients was small, we applied a nonparametric test. The significance of spectral parameters between pre- and post-stimulus epochs was evaluated using Wilcoxon's rank sum test with adjusted Bonferroni correction for multiple comparisons. The same test was also applied to the four subjects in whom both stimulation techniques were performed to assess the effect of bipolar vs. monopolar stimulation on spectral parameters. Statistical significance was set at *p* < 0.05 (uncorrected) and at *p* < 0.0125 (corrected).

## Results

**Figure 3:**
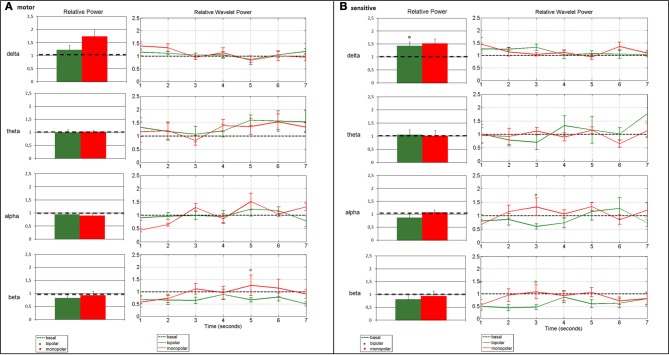
**Change in average spectral parameters after bipolar (patients nos. 1, 2, 4, 5, 6, 7) and monopolar (patients nos. 4, 5, 6, 7) stimulation compared to baseline in the motor (A) and the sensory (B) channels.** The left panels refer to the frequency analysis, and show the relative power in the four bands, calculated by assuming stationarity. The right panels refer to the time-frequency analysis, and show the relative wavelet power in the four bands, calculated by relaxing the stationarity assumption. Bars represent standard error (°*p* < 0.0125 bipolar vs. baseline).

All results are summarized in Figure 3. Outliers were excluded from the results (patient no. 3). The pattern of patient no. 3 was very different from the others and this could be derived from the poor quality of the signal because of many artifacts. Results were obtained before tumor removal.

### Bipolar stimulation vs. baseline

Frequency analysis of ECoG recordings showed a beta power decrease in the motor channel (*p* = 0.0476) and a significant delta power increase in the sensory channel (*p* = 0.0022) as compared to baseline. The time-frequency analysis confirmed the beta power decrease in the motor channel, which remained markedly but not significantly below the baseline value for the entire post-stimulus interval, as well as the power delta increase in the sensory channel, but was limited to the early portion of the interval. Additional transient alterations were observed on wavelet analysis: the delta power remained above the baseline value, even for the motor channel until about 3 s after the stimulus (Figure 3A). The theta rhythm varied in time and in frequency, without demonstrating a clear power trend. In the sensory channel, the alpha rhythm returned to baseline only after about 4.5 s, while in the beta range the power remained markedly below the baseline value for the entire post-stimulus interval in both channels (Figure 3B).

### Monopolar stimulation vs. baseline

Monopolar stimulation did not induce relevant changes in spectral parameters, except for delta power, which increased over motor (*p* = 0.0286) and sensory cortices (*p* = 0.0286), as compared to the baseline condition (Figures 3A,B). The time-frequency analysis showed that the increase in delta power after stimulus was confined to the early 3 s. The increase in alpha and beta powers occurred from 2 to 4 s after stimulus (Figure 3), without reaching statistical significance.

### Bipolar vs. monopolar stimulation

Comparison of the ECoG rhythms in the four patients in which both bipolar and monopolar stimulation were applied showed no significant changes in power as computed with the AR model. There was only a visible increase of power in the delta range and a decrease in the beta range in both channels compared to baseline. These trends were also detected with DWT analysis, which highlighted an increase of delta and a decrease of beta powers until about 4 s after the stimulus. No particular trend for alpha and theta relative powers was observed.

## Discussion

In this study we have quantified brain oscillatory activity by means of two different types of brain stimulation in awake patients. The novelty of the study resides in the demonstration of dynamic changes with a reliable time course after brain stimulation. The effect of these two different brain stimulation techniques was similar and involved all the frequencies that react with a different pattern. Because of the conservative nature of the Bonferroni correction for multiple comparisons, few results reached statistical significance; nevertheless the main modifications of delta and beta rhythms were anyway marked. An increase in delta activity over both the motor and the sensory cortices was noted in both modes of stimulation: the relative power in the delta band was increased compared to baseline (only the delta power of the sensory channel after bipolar stimulation reached statistical significance) and the time-frequency analysis suggested that these phenomena occur in the 4-s period following stimulation. In contrast, both modes of stimulation induced a decrease in beta activity: there was a marked deviation from baseline of the relative power in the beta band only in the motor channel during bipolar stimulation, and the time-frequency analysis indicated that a similar effect was present in the entire 7-s interval. Frequency and time-frequency analyses showed no significant difference between monopolar and bipolar stimulation. The relative power in the theta and the alpha bands was not significantly affected by stimulation, while the time-frequency analysis suggested some transient effect of stimulation, with a pattern characterized by a decrease in the early phase followed by a rebound. Taken together, our data indicate that both monopolar and bipolar stimulation can increase slow activity and decrease rapid activity over motor and sensory cortices.

The use of both methods reaches beyond cortical stimulation and is also applied for subcortical stimulation to identify and localize the corticospinal tract. The short-train technique leads to excitation of the pyramidal track; it is a safe and reliable way to map the primary motor cortex, and it is usually performed in anodal monopolar mode. Its main advantages over Penfield's technique are the lower risk of seizures and the potential use for continuous MEP monitoring. Bipolar stimulation provides a more focal electrical field than monopolar stimulation does. Hern et al. have demonstrated that monopolar anodal stimulation stimulates pyramidal cells directly; therefore, repetitive monopolar cortical stimulation induces repetitive excitation of the corticomotoneural tract (Kombos et al., 1999).

The novelty of our study resides also in the use of different ECoG analyses in awake patients never investigated, to our knowledge, during intraoperative monitoring. In order to quantify and measure the amount and the duration of brain oscillatory activity, ECoG powers after both modes of stimulation were evaluated using frequency and time-frequency analyses. Assumptions underlying the two methods (AR modeling for frequency analysis, discrete wavelet decomposition for time-frequency analysis) obviously differ, as does the meaning of the parameters they provide, which may explain the reason why both methodologies are applied. AR modeling, performed for the frequency analysis, provides quantitative information about the overall modifications of the power spectral bands in a 7-s period after stimulation and suggests possible processes related to cortical oscillation activity. However, because it assumes stationarity, it does not yield information about the time at which these modifications occur. Time-frequency analysis addresses this issue: monitoring the time course of spectral parameters adds to the information on the time interval at which a modification occurs.

Accuracy, safety and efficiency are all important considerations when setting up procedures for electrocortical stimulation. Compared to monopolar stimulation, the distribution of the electric field generated by bipolar stimulation may be more focal because the anode and cathode are very close, thus limiting current spreading. This is why it is essential to consider the differences in stimulus parameters in cortical mapping, particularly in epilepsy. Stimulating a cortical area can produce afterdischarges, sometimes followed by clinical seizures, whether or not that region causes spontaneous seizures (Lesser et al., 1984; Blume et al., 2004). To improve safety, extra care should be taken to avoid causing seizures during testing with electrical stimulation. During electrocortical stimulation mapping, ECoG is necessary to ensure safety and to detect afterdischarges. It serves to monitor the patient continuously for afterdischarge activity in the absence of physical signs of seizure, since afterdischarges may induce clinical seizures which can be very challenging for the operating room staff to manage. Afterdischarges can be seen at low current levels, that will be the limit in stimulation current that must not be exceeded to avoid inducing a clinical seizure, hence the importance of ECoG monitoring. Continuous ECoG monitoring also serves to verify stimulation by recording stimulation artifacts: without observing stimulus artifacts, it is impossible to know whether the absence of a response to stimulus was an accurate mapping or an indicator of technical failure. Since we analyzed ECoG epochs without afterdischarges, we are not able to describe the correlation between frequency band changes and afterdischarges. However a relationship can exists. Epileptiform activity such as afterdischarges may occur under conditions of deactivation (Pfurtscheller and Lopes da Silva, 1999). This might explain why seizure in some patients occurs during sleep. The occurrence of afterdischarges may be associated with greater power in lower frequency bands (Lesser et al., 2008). Therefore, an increase in delta activity such as after bipolar and monopolar stimulation can increase the probability of afterdischarges.

In our study, we did not observe any significant differences between the two modes of stimulation, although increased delta activity and decreased beta activity were noted in monopolar as compared to bipolar stimulation on the AR analysis, suggesting a more important effect on cortical oscillatory activity.

Of additional concern are such physiological factors as the subjects' own brain states, anxiety or nervousness and fear, attention to stimulation, expectation of pain, all of which might also induce power changes relative to baseline. Moreover, different stimulation settings could elicit different responses. Therefore, the investigation of the power of post-stimulation ECoG alone is insufficient to compare the effects of monopolar and bipolar stimulation.

The intraoperative use of electrical stimulation allows reliable identification of the sensorimotor region and constitutes a prerequisite for its anatomical and functional preservation. Previous studies have shown that various features of the ECoG power spectrum are modulated by movement-related activity (Pfurtscheller et al., 1996; Stancak and Pfurtscheller, 1996; Crone et al., 1998) or by imagined movements (Crone et al., 1998; Lopes da Silva and Pfurtscheller, 1999; Leuthardt et al., 2006). In a study (Gwinn et al., 2008) attempting to quantify the electrical changes in epilepsy patients undergoing functional mapping with an intracranial grid and strip electrodes, it was reported that Teager Energy measurements of ECoG can be used to compare activity levels in human brain tissue before and after stimulation. Specifically, 50 Hz stimulation in three patients caused a measurable increase in average energy, in frequencies >8 Hz, up to 10 s after stimulation.

The study focuses mainly on the analysis of rhythms related to the motor area, i.e., alpha and beta rhythms. However there is evidence that high-beta (20–30 Hz) and gamma (30–80 Hz) frequency bands are not clearly separated, but seem to be generated by the same underlying process (Steriade, 2006). The gamma activity, indeed, has been observed during a variety of functional activation tasks, including self-paced movement (Crone et al., 1998), auditory discrimination (Crone et al., 2001a) and word production tasks (Crone et al., 2001b). For example, although alpha and beta ERD have been observed over bilateral sensorimotor cortices during unilateral limb movements (Pfurtscheller and Klimesch, 1991) also gamma ERS has been observed over the contralateral sensorimotor cortex. Moreover, Sinai et al., showed that ECoG gamma had a more restricted distribution over perisylvian cortical regions known from lesion studies to be most critical for naming and other language functions (Sinai et al., 2005). Signals in gamma band are usually much lower in amplitude than the summed slow potentials related to postsynaptic activity (Buzsáki and Draguhn, 2004), therefore since ECoG has an excellent high frequency fidelity, it could be interesting in a future work to evaluate gamma changes in response to electrical stimulations.

The main conclusion of our study is that we observed an increase in the delta range for both the motor and the sensory channels, irrespective of the mode of stimulation, and that these phenomena occur in the 4-s period following stimulation. A decrease in the beta range was also observed with bipolar stimulation, and the time-frequency analysis indicated that a similar effect is present in the entire 7-s interval following stimulation. Moreover, frequency and time-frequency analyses of our datasets showed no significant difference between monopolar and bipolar stimulation, despite their different stimulus parameters of pulse duration, ISI and frequency.

The aim of our study was not to compare the two stimulation techniques but rather to describe their effect on brain oscillatory activity. Such a comparison would be faulty since the methods use different paradigms of stimulation. A major limitation of these data is the lack of a strict study protocol. No protocol was applied because the purpose of the procedure was not to compare the two stimulation techniques. We recorded ECoG signals during intraoperative monitoring before brain tumor surgery. Furthermore, the stimulation procedure did not follow an experimental design; instead, it was applied by the neurosurgeon in order to map the cortex.

The results of this study are preliminary; further studies on larger populations are therefore needed to confirm these results and to find a conclusion. We cannot define the possible epileptogenesis of either mode of stimulation and the data are insufficient to define their effect. Nonetheless, our data do add important insights into the patterns of oscillatory activity in the awake brain during stimulation and indicate the potential this new method of analysis holds in clinical intraoperative neurophysiology.

### Conflict of interest statement

The authors declare that the research was conducted in the absence of any commercial or financial relationships that could be construed as a potential conflict of interest.
